# The Effect of Mesenchymal Stromal Cells Derived From Endometriotic Lesions on Natural Killer Cell Function

**DOI:** 10.3389/fcell.2021.612714

**Published:** 2021-12-20

**Authors:** Fawaz Abomaray, Arnika Kathleen Wagner, Michael Chrobok, Åsa Ekblad-Nordberg, Sebastian Gidlöf, Evren Alici, Cecilia Götherström

**Affiliations:** ^1^ Division of Obstetrics and Gynecology, Department of Clinical Science, Intervention and Technology, Karolinska Institutet, Stockholm, Sweden; ^2^ Centre for Hematology and Regenerative Medicine, Department of Medicine, Karolinska Institutet, Stockholm, Sweden; ^3^ Department of Obstetrics and Gynecology, Stockholm South General Hospital, Stockholm, Sweden

**Keywords:** mesenchymal stromal cells, natural killer cells, endometriotic stromal cells, endometrial stromal cells, immunosuppressive, immunosurveillance, endometriosis

## Abstract

Endometriosis is an inflammatory disease that presents with ectopic endometriotic lesions. Reduced immunosurveillance of these lesions has been proposed to be playing a role in the pathology of endometriosis. Mesenchymal stromal cells (MSC) are found in ectopic lesions and may decrease immunosurveillance. In the present study, we examined if MSC contribute to reduced immunosurveillance through their immunosuppressive effects on natural killer (NK) cells. Stromal cells from endometriotic ovarian cysts (ESC_cyst_) and eutopic endometrium (ESC_endo_) of women with endometriosis and their conditioned medium were used in co-cultures with allogeneic peripheral blood NK cells. Following culture, NK cells were examined phenotypically for their expression of activating, inhibitory, maturation, and adhesion receptors and co-receptors, as well as the degranulation (CD107a) marker and the immunostimulatory (interferon-γ) and immunosuppressive (transforming growth factor beta 1 and interleukin-10) cytokines. Moreover, NK cell cytotoxicity was examined using chromium 51 release killing assays. There were no differences between ESC_cyst_ and ESC_endo_ regarding their effects on NK cell cytotoxicity in both conditioned medium and direct co-culture experiments. Additionally, there were no differences between ESC_cyst_ and ESC_endo_ regarding their impact on NK cells’ phenotype and degranulation in both conditioned medium and direct co-culture experiments. Although there were no differences found for DNAX accessory molecule-1 (DNAM-1) and NKp44, we found that the expression of the NK cell ligand CD155 that binds DNAM-1 and proliferating cell nuclear antigen (PCNA) that binds NKp44 was significantly less on ESC_cyst_ than on ESC_endo_. These findings were not supported by the results that the expression of the known and unknown ligands on ESC_cyst_ for DNAM-1 and NKp44 using chimeric proteins was not significantly different compared to ESC_endo_. In conclusion, the results suggest that ectopic MSC may not contribute to reduced immunosurveillance in endometriosis through their inhibitory effects on NK cells. This suggests that NK cell inhibition in the pelvic cavity of women with endometriosis develops due to other factors.

## Introduction

Endometriosis is a benign gynecological disease where the endometrium is found growing in ectopic sites, the pelvic cavity being the most common site (Vercellini et al., 2014). Endometriosis exhibits chronic inflammation, and its pathology is still not clearly understood (Vercellini et al., 2014). Approximately 10% of women of reproductive age are affected, and the main symptoms are pelvic pain and infertility (Kitawaki et al., 2002). The Sampson theory of ectopic implantation of the endometrium within the pelvic cavity by retrograde menstruation is the most widely accepted explanation for the origin of endometriosis. Although almost all women exhibit this phenomenon, only a small number develop the disease (Sourial et al., 2014), indicating that other factors may play a role in developing endometriosis. Various studies point to the notion that mesenchymal stromal cells (MSC) may contribute to the pathology (Mei et al., 2013; Mei et al., 2014; Nikoo et al., 2014) and that there is an immunological dysfunction (Schulke et al., 2009; Osuga et al., 2011; Kang et al., 2014) in the pelvic cavity leading to the survival and growth of ectopic tissue, which would normally be cleared away (Leavy, 2015).

MSC are mesodermal multipotent cells with known immunosuppressive effects found in different types of tissues (Fontaine et al., 2016; Abumaree et al., 2017). Moreover, we (Abomaray et al., 2017) and others (Kao et al., 2011) have found MSC in endometriotic lesions and the eutopic endometrium of women with endometriosis. The immunosuppressive effects of MSC on immune cell types such as macrophages (Abumaree et al., 2013) and natural killer cells (Spaggiari et al., 2008) have led to their use in clinical trials as a treatment for inflammatory diseases (Abumaree et al., 2017). However, we found that MSC may not be useful as a therapy for endometriosis, because they may directly support ectopic tissue growth (Abomaray et al., 2018). In addition, we found that in women with endometriosis, the inflammatory pelvic cavity may induce ectopic MSC to become more immunosuppressive than endometrial MSC (Abomaray et al., 2017). The ectopic MSC may be promoting alternative activation of macrophages (Abomaray et al., 2017), which is a finding that is in line with what is currently known about the macrophages present in the pelvic cavity, that they are predominantly immunosuppressive M2 macrophages (Bacci et al., 2009). Ectopic MSC may also influence the immunosurveillance of ectopic tissue through their effects on natural killer (NK) cells. Interestingly, NK cells have also been implicated in the pathology of endometriosis (Kang et al., 2014).

NK cells are lymphocytes involved in the innate immune system’s defense against cells that have been transformed by pathogens or malignancy (Chan et al., 2014). NK cells perform their cytotoxic functions *via* a detection system that involves various cell surface activating receptors such as NK group 2D (NKG2D) and DNAX accessory molecule-1 (DNAM-1) and inhibitory receptors such as NKG2A (Thiruchelvam et al., 2015). Moreover, killer cell immunoglobulin-like receptors, which are found on the surface of NK cells, are also involved in recognizing human leukocyte antigen (HLA) class I molecules on target cells to regulate NK cell cytotoxicity (Jeung et al., 2016). The extent of NK cell activation depends on the corresponding NK cell stress ligands and HLA proteins present on target cells, as well as the presence of cytokines in their microenvironment, such as interleukin-2 (IL-2) that is known to activate NK cells (Thiruchelvam et al., 2015; Kadri et al., 2016). NK cell activation induces their secretion of immunostimulatory cytokines, such as interferon-gamma (IFN-γ) (Vivier et al., 2008). In addition, it leads to their degranulation and release of CD107a-positive granules containing molecules, cytotoxic proteins, such as perforin, and the serine protease granzyme b that forms holes in the membranes of target cells and induces their subsequent apoptosis, respectively (Vivier et al., 2008).

Several studies have found that NK cells are inhibited in the pelvic cavity of women with endometriosis (Oosterlynck et al., 1991; Oosterlynck et al., 1992; Liu et al., 2018) and that the peritoneal fluid in women with endometriosis may contain certain factors that are inhibiting the function of NK cells (Oosterlynck et al., 1993; Kang et al., 2014). In addition, some studies have examined interactions between NK cells and ectopic MSC (Yu et al., 2016; Yang et al., 2017; Liu et al., 2018), suggesting that stromal cells may be involved in the inhibition of NK cells in the pelvic cavity in women with endometriosis. However, a comparison of the effects of stromal cells from endometriotic ovarian cysts (ESC_cyst_) and the endometrium (ESC_endo_) of women with endometriosis on NK cells *in vitro* has not been carried out previously. This comparison was made since ESC_cyst_ are most likely derived from ESC_endo_ based on the Sampson theory of retrograde menstruation, so we set out to examine the effects of the inflammatory pelvic cavity on ESC_cyst_ and hence their subsequent effects on NK cells. Therefore, this study compared the effects of ESC_cyst_ and ESC_endo_ of the same individuals on allogeneic peripheral blood NK cells *in vitro*. We hypothesized that ectopic MSC might be inhibiting the phenotype and cytotoxic functions of NK cells and contributing further to the overall reduced immunosurveillance of ectopic tissue in the pelvic cavity of women with endometriosis.

## Materials and Methods

### Human Tissue Samples

Two types of tissues were collected: 1) endometriotic ovarian cysts (ectopic endometrium) and 2) endometrium from the same women with endometriosis (eutopic endometrium). The endometriotic ovarian cysts and endometrium were collected from women aged between 31 and 42 years (mean ± SD, 36.3 ± 5.8 years, *n* = 4) undergoing laparoscopic surgery for confirmation and/or treatment of endometriosis. All women were histologically confirmed to have endometriosis by a pathologist. Only one woman underwent hormonal treatment. Two of the biopsies were from the proliferative phase, one was unknown, and one had amenorrhea. Ethical approval was obtained from The Regional Ethical Review Board in Stockholm (2013/1094-31/2), and informed oral and written consents were obtained from each participant.

### Isolation of Stromal Cells From Eutopic and Ectopic Endometrium

Stromal cells from human endometrial and endometriotic ovarian cyst tissues were isolated as previously described (Abomaray et al., 2017). Briefly, the tissues were digested to produce a single-cell suspension using 1 mg/ml collagenase type I (Sigma, Missouri, United States) diluted in Hank’s Balanced Salt Solution (Life Technologies, Paisley, United Kingdom) (90 min for endometriotic tissue and 30 min for endometrial tissue) at 37°C with regular shaking every 10 min. The digested tissues were filtered through 100- and 40-μm cell strainers (Corning, New York, United States). The cell suspension was washed with phosphate-buffered saline (PBS) (Life Technologies); then, the cell pellet was resuspended in complete growth medium containing Dulbecco’s Modified Essential Medium Low Glucose (DMEM-LG) (Life Technologies) + 10% MSC certified fetal calf serum (FCS) (Life Technologies) + 1% antibiotic and antimycotic (Life Technologies). The cells were cultured at 4,000 viable cells/cm^2^ in tissue culture flasks at 37°C with 5% CO_2_. When the cells reached 70%–90% confluency, they were trypsinized using 0.05% trypsin/EDTA (Life Technologies) and used for the experiments. To ensure that a pure population of cells was retrieved, the stromal cells were used at passages three to seven, as earlier passages of primary cells may be contaminated with other cell types. The stromal cells’ morphology, expression of MSC markers, their ability to form colonies, and differentiation into osteoblasts and adipocytes were characterized as previously described (Abomaray et al., 2017). The isolated cells formed colonies, had ≥95% expression for the MSC markers and ≤5% expression for the non-MSC markers, as well as differentiated into osteoblasts and adipocytes, altogether indicating that they are MSC (Abomaray et al., 2017).

### Isolation of Human Natural Killer Cells

Peripheral blood mononuclear cells (PBMCs) were isolated from buffy coats from healthy female donors using a Lymphoprep gradient separation according to the manufacturer’s instructions (Axis-Shield). Peripheral blood NK cells were isolated from the PBMCs (*n* = 30) using the NK Cell Isolation Kit (Miltenyi Biotech, Lund, Sweden) and a magnetic cell separation system (Miltenyi Biotech) as described previously (Abumaree et al., 2012). Isolated NK cells were cultured in complete growth medium containing stem cell growth medium (SCGM) (CellGenix, Freiburg, Germany) + 20% FCS + 500 IU/ml IL-2 (R&D Systems) for all experiments. The purity of the isolated NK cells was assessed by flow cytometry using the anti-CD56 and anti-CD3 monoclonal antibodies (Becton-Dickinson). Samples with a purity of ≥95% were used for experiments.

### Conditioned Medium Experiments

When the confluency for ESC_cyst_ and ESC_endo_ was ∼70%, the growth medium was discarded, the cells were washed twice with PBS, and then fresh growth medium was added. After 3 days, this conditioned medium was collected, centrifuged at 500*g* for 10 min to remove cellular debris, aliquoted, and frozen at −80°C. Following their isolation from PBMCs, NK cells were cultured in growth medium containing 50% of the aforementioned conditioned medium of ESC_cyst_ or ESC_endo_ and 50% SCGM growth medium containing SCGM + 20% FCS and 500 IU/ml IL-2 for 5 days. In addition, NK cells cultured in only complete SCGM growth medium were added as a control. Then, the NK cells were harvested for use in chromium 51 (^51^Cr) release assays with the non-adherent K562 cell line (ATCC) (chronic myelogenous leukemia cells that do not express HLA class I and therefore are efficiently lysed by NK cells) as target cells at 0.3:1, 1:1, 3:1, and 10:1 effector-to-target (*E*:*T*) cell ratios. The NK cells were also examined for their phenotype and degranulation using extracellular and intracellular flow cytometry.

### Direct Co-Culture Experiments

Following their isolation from PBMCs, NK cells were cultured in complete growth medium containing SCGM + 10% FCS + 500 IU/ml IL-2 for 2 days. Then, the NK cells were harvested for use in ^51^Cr release assays using ESC_cyst_ or ESC_endo_ as target cells in direct co-culture killing assay at 0.3:1, 1:1, 3:1, and 10:1 *E*:*T* ratios. The 3:1 cell ratio was selected and used to co-culture NK cells with ESC_cyst_ or ESC_endo_ as target cells, in order to examine NK cell phenotype and degranulation using extracellular and intracellular flow cytometry.

### Chromium 51 Release Assay

The ^51^Cr release assay was performed as previously published (Oosterlynck et al., 1991; Chrobok et al., 2019), but with slight modifications as described below. Briefly, the target cells (K562 cell line or ESC) were harvested and washed twice with PBS. For labeling with ^51^Cr, 100 µl of 1mCi (PerkinElmer) was added per 1 million K562 cells, or 15 µl of 1mCi + 15 µl Roswell Park Memorial Institute (RPMI) 1640 (GlutaMAX + 25 mM HEPES) (Life Technologies) were added per 0.15 × 10^5^ ESC. The target cells were incubated for 1 h at 37°C and 5% CO_2_, and they were gently resuspended regularly every 15 min in the case of ESC. The cells were washed twice with PBS, resuspended in RPMI 1640 + 10% FCS, and viable cells were counted. NK cells at densities of 1 × 10^3^, 3 × 10^3^, 9 × 10^3^, and 30 × 10^3^ cells in complete growth medium containing RPMI 1640 + 10% FCS were added per well in U-bottom 96-well plates (Corning), and 3,000 target cells were added to each well, so that *E*:*T* cell ratios of 0.3:1, 1:1, 3:1, and 10:1 were achieved. Negative control wells contained target cells in RPMI 1640 + 10% FCS for spontaneous release of ^51^Cr, and positive control wells contained 2 M hydrochloric acid for maximum lysis of ^51^Cr-labeled target cells and maximum release of ^51^Cr. All tests were performed in triplicates. The 96-well plates were incubated for 4 h at 37°C and 5% CO_2_, centrifuged at 300*g* for 5 min, and 70 µl of supernatant was transferred to micro-dilution tubes (SSIbio, United States) and analyzed in an Automatic Gamma Counter (PerkinElmer). The counts per minute were used to calculate the percentage killing of the target cells carried out by the effector NK cells using the standard formula:

% cytotoxicity=[(experimental−spontaneous release)/(maximum release−spontaneous release)×100]

### Extracellular Flow Cytometry

The NK cells or the ESC were labeled with Aqua-Vivid (live/dead cell marker, Invitrogen) for 20 min at 4°C, then washed with washing buffer (PBS + 2% FCS). For the conditioned medium experiments, the NK cells were labeled with the backbone receptors (found in all the panels) including CD3, CD14, CD19, CD56, and CD16; the activating receptors NKp30, NKp44, NKp46, NKG2D, NKG2C, and DNAM-1; the inhibitory receptor NKG2A; the maturation receptors including CD27, CD57, and cluster of differentiation molecule 11B (CD11b); and the adhesion receptors and co-receptors CD160, CD2, NKp80, CD2-like receptor activating cytotoxic cells (CRACC), CD161, sialic acid-binding Ig-like lectin 7 (Siglec 7), sialic acid-binding Ig-like lectin 9 (Siglec 9), anti-NK-T-B cell antigen (NTBA), and 2B4 (all monoclonal antibodies are from BD Biosciences or Biolegend: CD112 and CD155, see Table 1 for the colors of the fluorochromes) for 25 min at 4°C. For the direct co-culture experiments, the NK cells were labeled with CD3, CD16, and CD56, as well as the inhibitory receptors NKG2A, TIGIT, and CD96 and the activating receptor DNAM-1 (all monoclonal antibodies are from Becton-Dickinson or Biolegend: CD112 and CD155, see Table 1 for the colors of the fluorochromes) for 25 min at 4°C. For NK cell ligand staining, ESC_cyst_ and ESC_endo_ were labeled with E-cadherin (E-cad), N-cadherin (N-cad), CD155, CD112, MHC class I polypeptide-related sequence A or B (MIC-AB), human leukocyte antigen ABC (HLA-ABC), proliferating cell nuclear antigen (PCNA), and human leukocyte antigen C (HLA-C) (all monoclonal antibodies are from Becton-Dickinson, see Table 1 for the colors of the fluorochromes) for 25 min at 4°C. All the mastermixes containing the antibodies were prepared in brilliant staining buffer (Becton-Dickinson). For chimeric protein staining, ESC_cyst_ and ESC_endo_ were labeled with chimeric proteins consisting of human IgG fused to either NKG2D, NKp30, NKp44, NKp46, or DNAM-1 for 25 min at 4°C (all chimeric proteins are from R&D, see Table 1), then washed once with a washing buffer, before being labeled with a PE-conjugated secondary antibody for 25 min at 4°C (the secondary antibody was used alone as a control). The NK cells or the ESC were washed once with PBS, then resuspended in PBS and fixed with paraformaldehyde (PFA) (Sigma), before being acquired with the LSR Fortessa (BD) or CytoFLEX S Flow Cytometers (Beckman Coulter), respectively. The fluorescence minus one (FMO) controls, unstained cells, and backbone receptors were used to set the gates, and unstained cells and beads were used to compensate the fluorochromes (Becton-Dickinson). The data were analyzed using the software FlowJo (version 10.1r5, Tree Star, Inc. Ashland, United States).

**TABLE 1:** Antibodies and their fluorochromes used in this study.

Monoclonal/chimeric antibody	Fluorochrome
CD3	V450/BV421/BV510
CD14	V500
CD19	V500
CD56	BV605 and BV605
CD16	BV711/BV737
NKp30	Alexa647
NKp44	Alexa647
NKp46	PC7
NKG2D	PE-Cy7
NKG2C	Alexa488
DNAM-1	PE/BUV395
NKG2A	VioBright FITC/FITC
CD27	PE-CF594
CD57	PC-CF594
CD11b	Alexa700
CD160	PCPC5.5
CD2	APC-H7
NKp80	APC
CRACC	PE
CD161	PerCP-Cy5.5
Siglec-7	PE-Vio770
Siglec-9	FITC
NTBA	PE
2B4	PCPC5.5
CD107a	FITC/PE-Cy7/APC-Cy7
IFN-γ	APC
CD96	BV421
TIGIT	PE Dazzle 594
LAP1 (TGFβ1)	PE-Cy7
IL-10	Alexa Fluor 647
CD112	PE-Cy7
CD155	PerCP-Cy5.5
E-cad	FITC
N-cad	Alexa Fluor 647
MIC-AB	FITC
PCNA	Alexa Fluor 647
HLA-ABC	APC-Cy7
HLA-C	PE
Against DNAM-1 NK cell ligands	Not Applicable
Against NKG2D NK cell ligands	Not Applicable
Against NKp30 NK cell ligands	Not Applicable
Against NKp44 NK cell ligands	Not Applicable
Against NKp46 NK cell ligands	Not Applicable
Secondary antibody	PE

DNAM-1, DNAX accessory molecule-1; CD11b, cluster of differentiation molecule 11B; CRACC, CD2-like receptor-activating cytotoxic cell; Siglec 7, sialic acid-binding Ig-like lectin 7; Siglec 9, sialic acid-binding Ig-like lectin 9; NTBA, anti-NK-T-B cell antigen; IFN-γ, interferon-gamma; TIGIT, T cell immunoreceptor with Ig and ITIM domains; TGFβ1, transforming growth factor beta 1; IL-10, interleukin-10; E-cad, E-cadherin; N-Cad, N-cadherin; MIC-AB, MHC class I polypeptide-related sequence A or B; PCNA, proliferating cell nuclear antigen; HLA-ABC, human leukocyte antigen ABC; HLA-C, human leukocyte antigen C.

### Intracellular Flow Cytometry

The NK cells were incubated with target cells (ESC_cyst_ and ESC_endo_) or the K562 cell line (positive control) or treated with phorbol myristate acetate (PMA) (Sigma) and ionomycin (positive control, Sigma) to induce cellular activation. The CD107a FITC antibody (Becton-Dickinson) was added, and the cells were incubated for 1 h at 37°C and 5% CO_2_. Then, GolgiStop (BD Biosciences, California, United States) was added, and the cells were incubated for an additional 3 h at 37°C and 5% CO_2_; after which, the cells were washed twice with PBS; stained with CD3, CD16, and CD56 prepared in BD Brilliant Stain Buffer; and incubated for 25 min at 4°C. The cells were washed with PBS and fixed and permeabilized using BD cytofix/cytoperm (BD Biosciences) for 10 min at room temperature (RT). The cells were washed with 1X Perm/Wash (BD Biosciences), then stained for IFN-γ (Becton-Dickinson) for the conditioned medium experiments or stained with IFN-γ, interleukin-10 (IL-10), and transforming growth factor beta 1 (TGFβ1/LAP1) (all Becton-Dickinson) for the direct co-culture experiments, prepared in 1X Perm/Wash, and incubated for 25 min at RT. The NK cells were then washed twice with 1X Perm/Wash and PBS, then resuspended in PBS and fixed with PFA, before being acquired on the CytoFLEX S Flow Cytometer (Beckman Coulter). FMO controls and unstimulated NK cells were used to set the gates. Unstained cells and beads were used for compensation of the fluorochromes. The data were analyzed using the software FlowJo.

### Statistical Analysis

All statistical analyses were performed using GraphPad Prism 6. When the data were normally distributed, the means were analyzed with Student’s *t*-test, and when it was not normally distributed, the medians were analyzed with the Mann–Whitney test. All values are shown as the mean ± standard deviations (SD). For the study, *n* refers to the number of biological replicates. Results were considered to be statistically significant if *p* < 0.05.

## Results

### The Effect of Secreted Factors From ESC on NK Cell Phenotype

**FIGURE 1:**
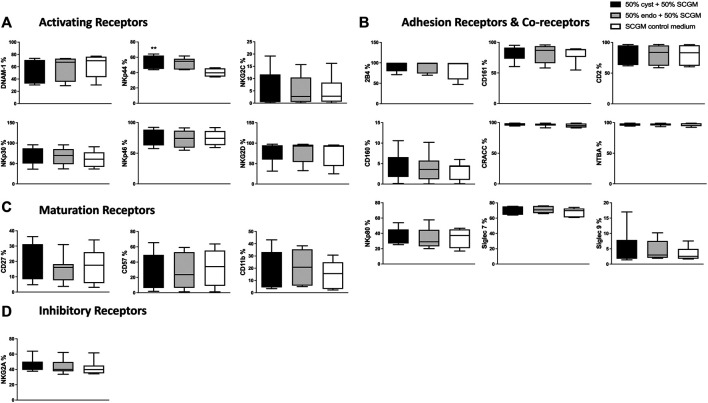
The phenotype of NK cells following culture in the conditioned medium of ESC_cyst_ or ESC_endo_ or in SCGM control medium for 5 days was analyzed using flow cytometry. There were no significant differences for the percentage expression between ESC_cyst_- and ESC_endo_-treated NK cells for all of the activating receptors **(A)**, adhesion receptors and co-receptors **(B)**, and maturation **(C)** and inhibitory **(D)** receptors. The conditioned medium was 50% derived from ESC_cyst_ or ESC_endo_ and 50% complete SCGM growth medium or 100% SCGM control medium. Eight independent experiments (*n* = 3 biological replicates) were carried out. Mean ± SD. NK, natural killer; ESC_cyst_, endometriotic ovarian cysts; ESC_endo_, eutopic endometrium; SCGM, stem cell growth medium.

We set out to determine the effects of ESC_cyst_ and ESC_endo_, respectively, on the expression of activating receptors, adhesion receptors and co-receptors, maturation markers, and inhibitory receptors of NK cells. We chose healthy donor peripheral blood NK cells as they have not been in the inflammatory environment in the pelvic cavity that is described in the endometriosis setting. NK cells were isolated and cultured for 5 days in the presence of IL-2 in the conditioned medium of ESC_cyst_ or ESC_endo_ or control medium as described in *Materials and Methods* section. After 5 days, the NK cells were analyzed by flow cytometry for surface expression of activating and inhibitory receptors, as well as maturation and adhesion receptors and co-receptors. There was no significant the asteriks in NKp44 plot should be above the SCGM control box (white), as that differed from the 2 experimental samples. These did not differe form each other, as described in this section of the Results. Difference in the percentage expression of any of the analyzed receptors between the two groups that were cultured with conditioned medium from either ESC_endo_ or ESC_cyst_ (Figure 1A–D). Furthermore, there was no significant difference in the expression levels (median fluorescence intensity, MFI) (Supplementary Figure S1A–D).

### The Effect of Secreted Factors From ESC on NK Cell Functionality

**FIGURE 2:**
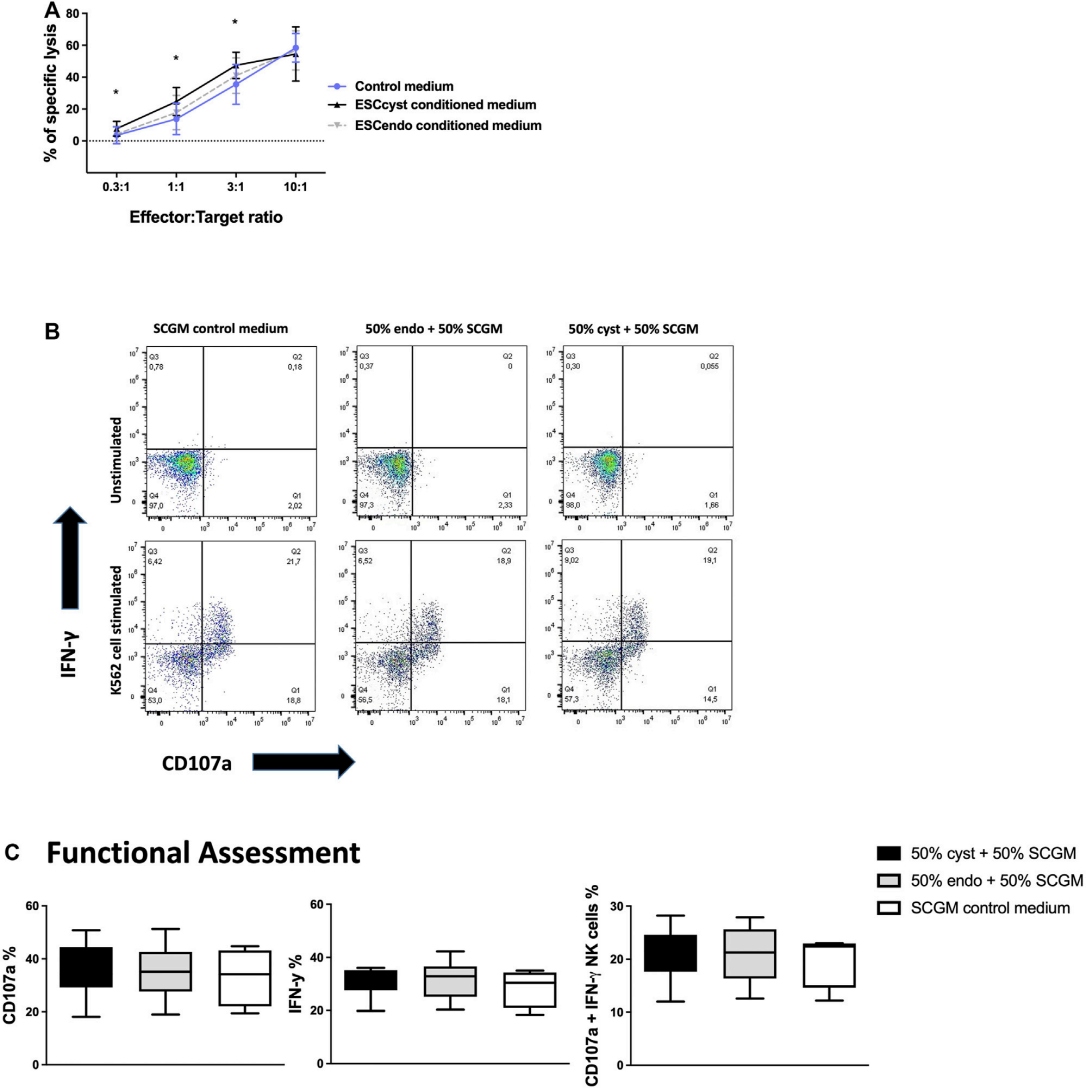
Chromium 51 (^51^Cr) release assays for NK cell killing of the K562 cell line following their culture in the conditioned medium of ESC_cyst_ or ESC_endo_ or control medium for 5 days **(A)**, as well as degranulation of NK cells following culture in the conditioned medium of ESC_cyst_ or ESC_endo_ or control medium for 5 days analyzed using flow cytometry **(B, C)**. There was a significant (*p* < 0.05) increase in NK cell (effector) killing of the K562 cell line (target) following their treatment with the conditioned medium of ESC_cyst_ compared to ESC_endo_ at only the 0.3:1, 1:1, and 3:1 cell ratios **(A)**. Representative dot plots showing interferon-gamma (IFN-γ) versus CD107a expression for the NK cells cultured in the conditioned medium of ESC_cyst_ or ESC_endo_ or control medium for 5 days, then either unstimulated or stimulated with K562 cells **(B)**. There were no significant differences for the percentage expression between ESC_cyst_, ESC_endo_, and SCGM-treated NK cells following culture with the K562 cell line for the degranulation marker CD107a or the immunostimulatory cytokine IFN-γ and for the percentage of NK cells that degranulated and expressed IFN-γ **(C)**. The conditioned medium was 50% derived from ESC_cyst_ or ESC_endo_ and 50% complete SCGM growth medium or 100% SCGM control medium. Seven **(A)** independent experiments (*n* = 4 biological replicates) were carried out in triplicate. Eight **(B, C)** independent experiments (*n* = 3 biological replicates) were carried out. Mean ± SD. CM, conditioned medium.

In order to determine the effects of ESC_cyst_ and ESC_endo_, respectively, on NK cell functional responses, we cultured healthy donor peripheral blood NK cells in the conditioned medium of ESC_cyst_ or ESC_endo_ in the presence of IL-2. After 5 days, NK cells were used in functional assays to determine whether the soluble factors secreted by ESC had a potential inhibitory or activating effect on NK cells. We used a standard 4-h ^51^Cr release assay against K562 cells, a myelogenous leukemia cell line that is killed efficiently by healthy donor NK cells. We ensured that the assay worked by utilizing positive and negative controls, which had maximum and minimum ^51^Cr release, respectively. NK cells that had been cultured in the conditioned medium from ESC_cyst_ had a small but significant (*p* < 0.05) increase in killing K562 target cells compared to NK cells that had been cultured in the conditioned medium from ESC_endo_. This was seen at the 0.3:1, 1:1, and 3:1 cell ratios, but there was no difference for the 10:1 cell ratio (Figure 2A). Although secreted factors of ESC_cyst_ and ESC_endo_ had similar effects on allogeneic NK cell cytotoxicity, we asked if these may impose different effects on NK cell degranulation and cytokine production. NK cells were cultured in the conditioned media as before and then used in a flow cytometry-based functionality assay, where K562 cells were used as stimulating cells. Degranulation was assessed by CD107a surface expression, while cytokine production was measured by intracellular staining of the pro-inflammatory cytokine IFN-γ (Figure 2B, C). NK cells cultured in conditioned media responded to stimulation by K562 target cells (Supplementary Figure S2). However, there was no significant difference when NK cells were cultured in the conditioned medium from ESC_cyst_ compared to the NK cells cultured in the conditioned medium from ESC_endo_ (Figure 2C **)**. Taken together, the results indicate that there is only a minor increase in cytotoxicity when NK cells were cultured in the conditioned medium from ESC_cyst_ compared to those cultured in the conditioned medium from ESC_endo_.

### The Killing of ESC by NK Cells

**FIGURE 3:**
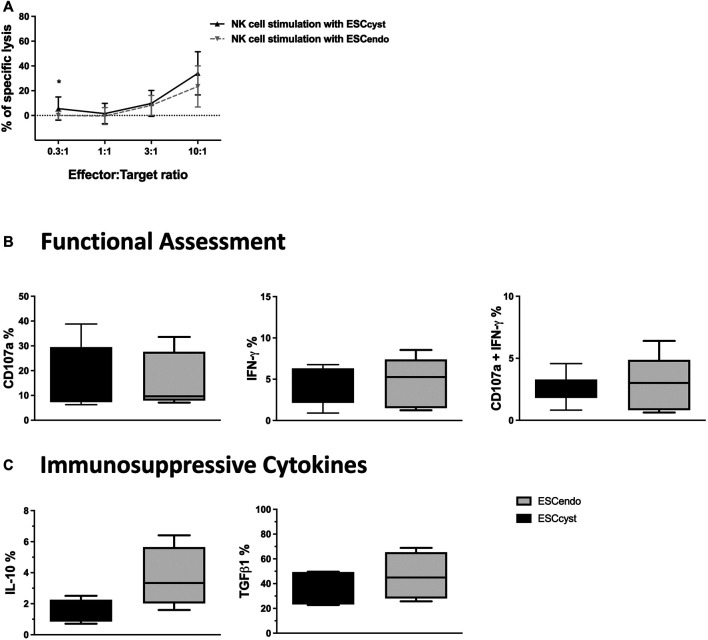
Chromium 51 (^51^Cr) release assays for NK cell killing of ESC_cyst_ or ESC_endo_ **(A)**, as well as degranulation of NK cells when stimulated with ESC_cyst_ or ESC_endo_ for 4 h at a 3:1 cell ratio analyzed using flow cytometry **(B, C)**. NK cell (effector) killing of ESC_cyst_ or ESC_endo_ (target) was not significantly different for all the cell ratios, except the 0.3:1 cell ratio **(A)**. There were no significant differences between ESC_cyst_- and ESC_endo_-stimulated NK cells for the percentage expression of the degranulation marker CD107a or IFN-γ and for the percentage of NK cells that degranulated and expressed IFN-γ **(B)**. The percentage expression of the immunosuppressive cytokines transforming growth factor beta 1 (TGFβ1) and interleukin-10 (IL-10) in the NK cells that were stimulated with ESC_cyst_ was not different from the NK cells stimulated with ESC_endo_ **(C)**. Five **(A)** independent experiments (*n* = 4 biological replicates) were carried out in triplicate. Six–eight **(B, C)** independent experiments (*n* = 4 biological replicates) were carried out. Mean ± SD.

NK cells are not only known to kill transformed and infected cells but can also clear the body of activated and proliferating cells to maintain homeostasis. We asked whether healthy donor peripheral blood NK cells would kill ESC from ectopic or eutopic endometrium isolated from the same individuals with endometriosis. To assess this, we used ESC_cyst_ or ESC_endo_ as target cells in the functional assays described above. For direct cytotoxicity, ESC_cyst_ or ESC_endo_ were labeled with ^51^Cr as described in the *Materials and Methods* section and then used as target cells in a standard 4-h killing assay. NK cell cytotoxicity was similar for ESC_cyst_ compared to ESC_endo_, in all the cell ratios used, except for the 0.3:1 cell ratio that had a significant difference (*p* < 0.05) (Figure 3A). In order to assess NK cell responses towards ESC, we assessed degranulation and cytokine production of NK cells as described before. In addition to IFN-γ, we also stained for the anti-inflammatory cytokines TGFβ1 and IL-10 in this assay. NK cells that were stimulated with the K562 cell line as a positive control showed that the percentage of NK cells that expressed the degranulation marker CD107a and IFN-γ was significantly (*p* < 0.05) greater compared to unstimulated NK cells (Supplementary Figure S3A). The expression of CD107a and IFN-γ of NK cells stimulated by ESC_cyst_ was not significantly different from the NK cells stimulated by ESC_endo_ (Figure 3B **)**. Furthermore, the expression of the immunosuppressive cytokines TGFβ1 and IL-10 did not differ significantly between stimulation with ESC_cyst_ or ESC_endo_ (Figure 3C). Moreover, the expression levels did not show significant differences for any functional markers (Supplementary Figure S3B, C). Taken together, the results indicate that peripheral blood NK cells do not exert different functional responses when stimulated with ESC from either ectopic or eutopic endometrium.

### The Comparison of NK Cell Ligand Expression in ESC_cyst_ and ESC_endo_

**FIGURE 4:**
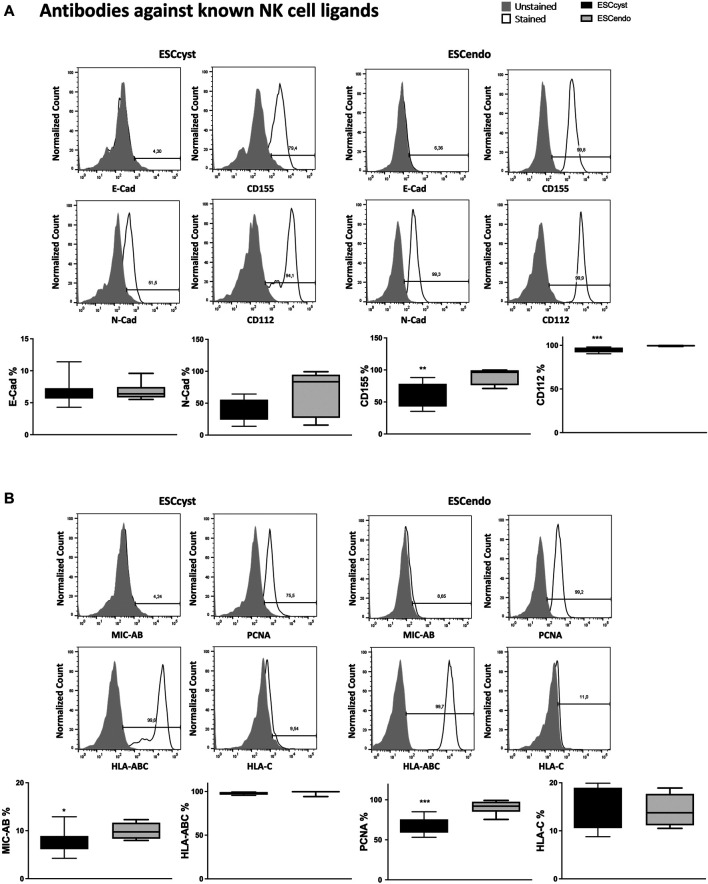
The expression of known NK cell ligands on ESC_cyst_ and ESC_endo_ was examined using monoclonal antibodies, with representative histograms. There were no differences in the percentage expression of all the NK cell ligands using the monoclonal antibodies between ESC_cyst_ and ESC_endo_, except for CD112, CD155, proliferating cell nuclear antigen (PCNA), and MHC class I polypeptide-related sequence **A** or **B** (MIC-AB), which were significantly (*p* < 0.05) different. Eight independent experiments (*n* = 4 biological replicates) were carried out. Mean ± SD.

The expression of NK cell ligands on ESC could activate or inhibit NK cells (Thiruchelvam et al., 2015); therefore, we examined their expression on ESC_endo_ and ESC_cyst_. We stained the ESC with antibodies specific for known ligands of typical NK cell receptors (Figure 4) and chimeric proteins consisting of the extracellular domains of activating NK cell receptors, for detection of unknown ligands to these receptors (Supplementary Figure S4A, B). The ESC express N-cad, CD155, CD112, HLA-AB, and PCNA (Figure 4). The ligands for inhibitory NK cell receptors, N-cad, and HLA-ABC did not show a difference in surface expression between ESC_cyst_ and ESC_endo_ (Figure 4). However, some ligands for activating NK cell receptors were expressed lower on ESC_cyst_ compared to ESC_endo_ (Figure 4). The adhesion molecules CD155 and CD112 that bind to the activating NK cell receptor DNAM-1 were both down-regulated on ESC_cyst_ compared to ESC_endo_ (Figure 4). Both molecules can also be recognized by the inhibitory receptors TIGIT and CD96. In addition, MIC-AB (recognized by NKG2D) and PCNA (recognized by NKp30 and NKp44) (Rosental et al., 2011; Kundu et al., 2019) were expressed lower on ESC_cyst_ compared to ESC_endo_ (Figure 4). However, when staining with chimeric proteins, we could not see a difference in the proportion of cells expressing the ligands as well as the expression levels of the ligands (Supplementary Figure S4A, B). Taken together, the results indicate that there is an expression of ligands that could trigger NK cells differently in ESC_cyst_ compared to ESC_endo_.

### DNAM-1 and TIGIT on NK Cells May Interact With CD155 and CD112 on ESC

**FIGURE 5:**
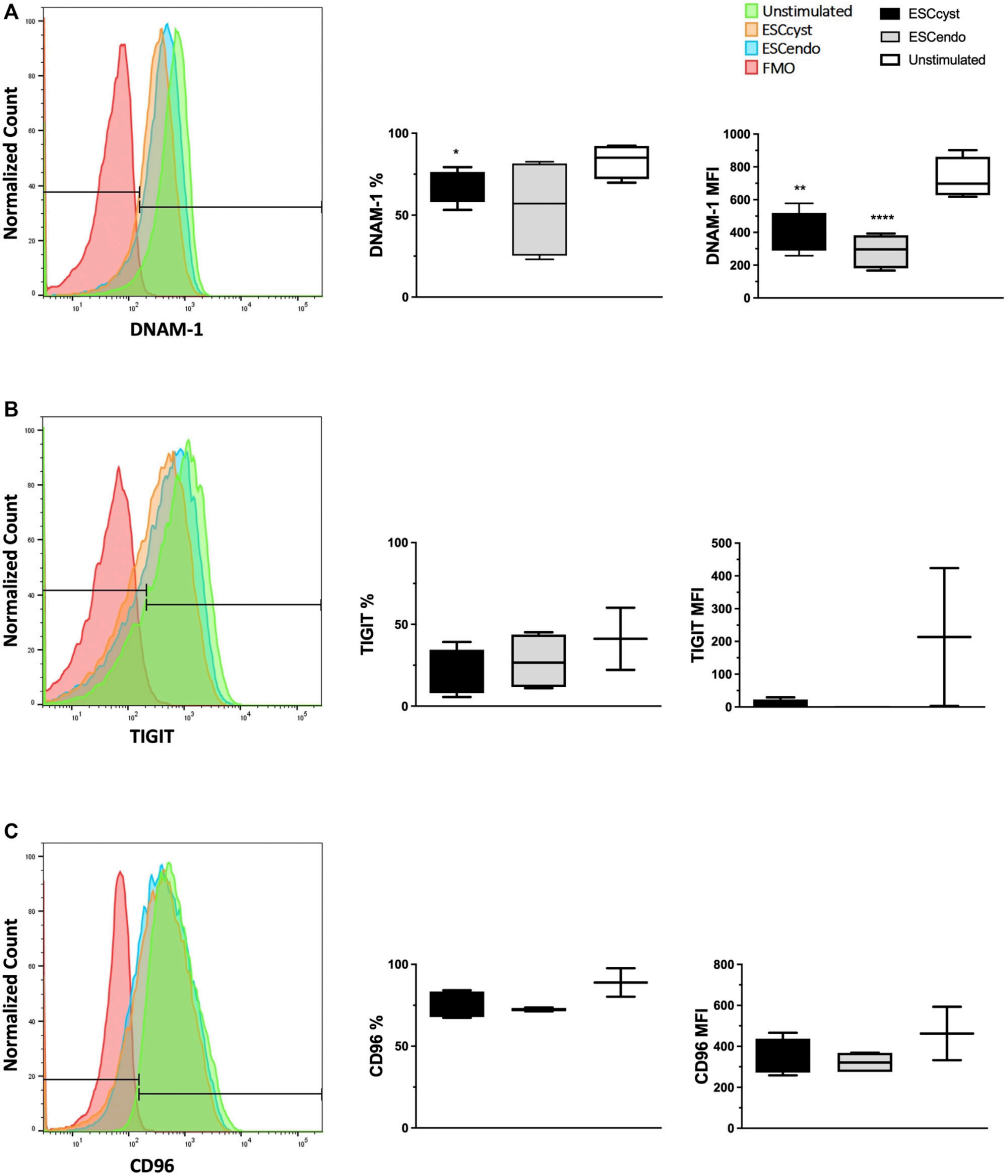
Receptor expression of NK cells stimulated with ESC_cyst_ or ESC_endo_ for 4 h at a 3:1 cell ratio was analyzed using flow cytometry, showing representative histograms. The percentage expression and expression levels of the surface activating receptor DNAM-1 in the NK cells stimulated with ESC_cyst_ were not significantly different compared to the NK cells that were stimulated with ESC_endo_ **(A)**. The NK cells that were stimulated with ESC_cyst_ showed no difference for the percentage expression and the expression levels compared to the NK cells that were stimulated with ESC_endo_, for the surface inhibitory receptors TIGIT and CD96 **(B, C)**. Unstimulated NK cells were incubated with medium only. The fluorescence minus one (FMO) controls contained all of the NK cell markers examined, except each marker presented here. Six–eight independent experiments (*n* = 4 biological replicates) were carried out. Mean ± SD.

Since both CD112 and CD155, the common ligands for DNAM-1, TIGIT, and CD96, were differentially expressed on ESC, we asked whether there was any down-regulation of these NK cell receptors during the interaction of NK cells with ESC. Therefore, we stimulated healthy donor peripheral blood NK cells with ESC_cyst_ or ESC_endo_ for 4 h and measured the levels of DNAM-1, TIGIT, and CD96 after the assay by flow cytometry. Compared to unstimulated NK cells that had been incubated with medium only, the expression levels of DNAM-1 (Figure 5A) and TIGIT (Figure 5B), but not CD96 (Figure 5C), decreased on NK cells stimulated with ESC. While the expression of DNAM-1 decreased more for ESC_endo_ than for ESC_cyst_, the opposite was observed for TIGIT. The expression of another inhibitory receptor, NKG2A, was not changed upon stimulation with ESC (Supplementary Figure S5). These results could indicate that NK cells interact with ESC *via* the receptors DNAM-1 and TIGIT on NK cells, with the ligands CD155 and CD1112 on ESC. This interaction could favor the activating receptor DNAM-1 when NK cells are recognizing ESC_endo_, while it could be biased towards TIGIT when they interact with ESC_cyst_.

## Discussion

Currently, the cause of reduced immunosurveillance of endometriotic lesions in the pelvic cavity of women with endometriosis is unknown. One of the components of this reduced immunosurveillance is the inhibition of NK cells (Oosterlynck et al., 1991; Oosterlynck et al., 1992; Liu et al., 2018; Xu, 2019). Therefore, in the present study, we investigated if ESC_cyst_ may also be involved in NK cell-mediated inhibition, because we previously found that they may possess immunosuppressive properties (Abomaray et al., 2017). However, the findings from the present study suggest that ESC from endometriotic ovarian cysts may not impose a more significant inhibition on NK cells compared to ESC from the endometrium.

We found that treating healthy allogeneic NK cells with conditioned medium from ESC_cyst_ and ESC_endo_ did not result in a substantial difference in NK cell cytotoxicity. In line with this, the phenotype of NK cells treated with conditioned medium from ESC_cyst_ was similar to that of NK cells treated with conditioned medium from ESC_endo_; there were no significant differences in the proportion of cells expressing the receptors and the expression levels of the activating, inhibitory, maturation, or adhesion receptors and co-receptors. Moreover, secreted factors of ESC_cyst_ or ESC_endo_ did not induce or dampen the functional responses of NK cells against the standard NK-target cell line K562. There have been no studies comparing the conditioned medium of ESC_cyst_ and ESC_endo_ on the phenotype of NK cells to the best of our knowledge. However, there have been studies examining the effects of conditioned medium of ESC on NK cell cytotoxicity. Previously, Somigliana et al. (1996b) examined the effect of conditioned medium from ESC_endo_ from healthy female volunteers (ESC_endohv_) on the cytotoxicity of NK cells isolated from healthy female volunteers using ESC_endohv_ as target cells and found that NK cell cytotoxicity was significantly inhibited. The same group compared the effects of conditioned medium from ESC_endo_ to ESC_endohv_ on the cytotoxicity of NK cells isolated from healthy female volunteers using ESC_endohv_ as target cells, and they found that NK cell cytotoxicity was significantly inhibited by conditioned medium from ESC_endo_ compared to ESC_endohv_ (Somigliana et al., 1996a). At present, we do not know why our results do not show a reduction of NK cell cytotoxicity when cultured in conditioned medium from ESC. The studies by Somigliana et al. (1996a; 1996b) compare ESC_endo_ to ESC_endohv_, and this may introduce inter-individual variations in terms of the immunological microenvironment and endocrine factors. Furthermore, healthy eutopic endometrium serves as an inappropriate model for studying NK cell responses in endometriosis, as ESC from ectopic endometriotic lesions may have a significantly different behavior. By contrast, in the current study, we compared NK cells cultured in conditioned medium from ESC_endo_ or ESC_cyst_ that were isolated from different sites of the same women with endometriosis. This setup allows us to study the effects of ESC from different microenvironments, but from the same individuals, on NK cell functions. One potential pitfall of this setup may be the fact that factors secreted into the conditioned medium of ESC_cyst_ may not have been present at a significant concentration to have an evident effect on NK cell cytotoxicity since they may have been short-lived as many cytokines are (Zhou et al., 2010). Therefore, we used ESC from the different sites as target cells to stimulate the NK cells to compare the effects of direct contact with ESC_cyst_ and ESC_endo_ on NK cell cytotoxicity.

Similarly, as for the experiments using the conditioned medium, the cytotoxicity of the NK cells was not different towards ESC_endo_ or ESC_cyst_. Previously, Oosterlynck et al. (1991; 1992) found that NK cells from the peripheral blood and peritoneal fluid of women with endometriosis have significantly reduced cytotoxicity against the K562 cell line and autologous ESC_endo_ compared to NK cells from women without endometriosis. A similar study found that NK cells from the peripheral blood and peritoneal fluid of women with endometriosis had significantly reduced cytotoxicity against the K562 cell line compared to women without endometriosis (Wu et al., 2000).

NK cells recognize target cells *via* an array of germline-encoded activating and inhibitory receptors. The integration of positive and negative signals determines whether the NK cell becomes activated and if the target cell is killed (DOI: 10.1038/ni1581, L Lanier: up on the tightrope: natural killer cell activation and inhibition). We therefore set out to identify the ligands on ESC that may contribute to the recognition by NK cells. We used a panel of antibodies for inhibitory and activating ligands, as well as chimeric proteins that bind the ligands *via* the natural receptor–ligand interaction. We found that ESC express several ligands for activating and inhibitory NK cell receptors. However, only the CD155 and CD112 ligands for the NK cell receptors DNAM-1, TIGIT, and CD96 differed in their expression between ESC_endo_ and ESC_cyst_. Therefore, we speculate that one or several NK cell receptors of this receptor–ligand network may interact with the ligands on ESC. We saw receptor down-regulation after 4 h of co-culture of DNAM-1 and TIGIT, but not of CD96. This down-regulation may indicate an interaction with the ligands on ESC.

In addition to DNAM-1 and TIGIT, the expression of PCNA, a ligand for NKp30 and NKp44, was significantly less on ESC_cyst_ than on ESC_endo_, which is known to inhibit NK cell function through the NKp44 receptor (Kundu et al., 2019). Interestingly, Yu et al. (2016) have shown that ESC from ectopic endometrium secrete high amounts of IL-15, which leads to a down-regulation of NKp44 and NKG2D on NK cells. Taken together, these combined findings may hint towards the regulation of DNAM-1, TIGIT, NKp44, and NKG2D on NK cells by ESC. However, we could neither detect high levels of the NKG2D-ligands MIC-A/B on ESC nor was there a difference between ESC_endo_ and ESC_cyst_ in the expression of MIC-A/B. The same group used NK cells cultured alone or in indirect transwell co-cultures with ESC from ectopic lesions, and then, the NK cells were kept in direct co-culture with ESC from ectopic lesions in a different cytotoxicity assay (lactate dehydrogenase assay) (Yang et al., 2017). They found no significant differences in NK cell cytotoxicity between NK cells cultured alone and NK cells indirectly co-cultured with ESC from ectopic lesions (Yang et al., 2017), similar to our results with conditioned medium from ESC_endo_ and ESC_cyst_. They further employed a co-culture system where ESC_cyst_ and macrophages were directly co-cultured with NK cells and compared the results to NK cells cultured alone (Yang et al., 2017). It was found that NK cell cytotoxicity was significantly reduced, and it was determined that it may be due to the secretion of the immunosuppressive molecules transforming growth factor beta 1 and IL-10 from both ESC_cyst_ and macrophages (Yang et al., 2017). However, it seems that the effects on the NK cells are mostly mediated through macrophages and not ESC_cyst_, since NK cell cytotoxicity was not reduced following co-culture with only ESC_cyst_ (Yang et al., 2017). In another study, NK cells were cultured with ESC_endohv_ or ectopic ESC; it was found that the NK cells significantly enhanced their expression of the immunosuppressive enzyme indoleamine 2,3-dioxygenase (IDO), especially following culture with ectopic ESC, and the effect was partly due to ESC-derived TGFβ1 (Liu et al., 2018). Moreover, when the NK cells were in turn cultured with ectopic ESC, their cytotoxicity was significantly inhibited, and this was attributed to their expression of IDO (Liu et al., 2018). As all the studies mentioned above, we have used NK cells from healthy female volunteers. The use of healthy NK cells that have previously not been in the environment of the inflammatory pelvic cavity described in the endometriosis setting allows one to examine if ESC_cyst_ are more immunosuppressive, at least through their effects on the NK cells, than ESC_endo_, due to this inflammatory environment found in the pelvic cavity of women with endometriosis. However, future studies could investigate the effects of ESC on NK cells isolated from women with endometriosis, when studying MSC immunomodulation of NK cells in endometriosis.

Although our study used stromal cells from a limited number of donors, the results were consistent between the four women with endometriosis that we studied. Moreover, to our knowledge, this is the first study directly comparing the effects of ESC_cyst_ and ESC_endo_ from the same women with endometriosis on with endometriosis on previously unaffected healthy donor NK cells. Our results suggest that ectopic MSC may not be contributing to the reduced immunosurveillance in the pelvic cavity of women with endometriosis through their effects on NK cells.

In summary, inhibition of NK cell function in the pelvic cavity in women with endometriosis may not be due to direct interactions with ectopic MSC. This finding suggests that other factors present in the pelvic cavity may be involved in the inhibition of NK cells. However, a future study utilizing a similar co-culture system, but with NK cells from women with endometriosis, should be carried out, in order to confirm our findings.

## Data Availability

The original contributions presented in the study are included in the article/Supplementary Material; further inquiries can be directed to the corresponding author.
