# Supplementary material for: The Effect of Mesenchymal Stromal Cells Derived From Endometriotic Lesions on Natural Killer Cell Function
Source: Front Cell Dev Biol. 2021 Dec 20;9:612714. doi: 10.3389/fcell.2021.612714 (PMC8722454; doi:10.3389/fcell.2021.612714)
Supplement: Supplementary file 1 [file DataSheet1.docx]

Supplementary Material

## Supplementary Figures


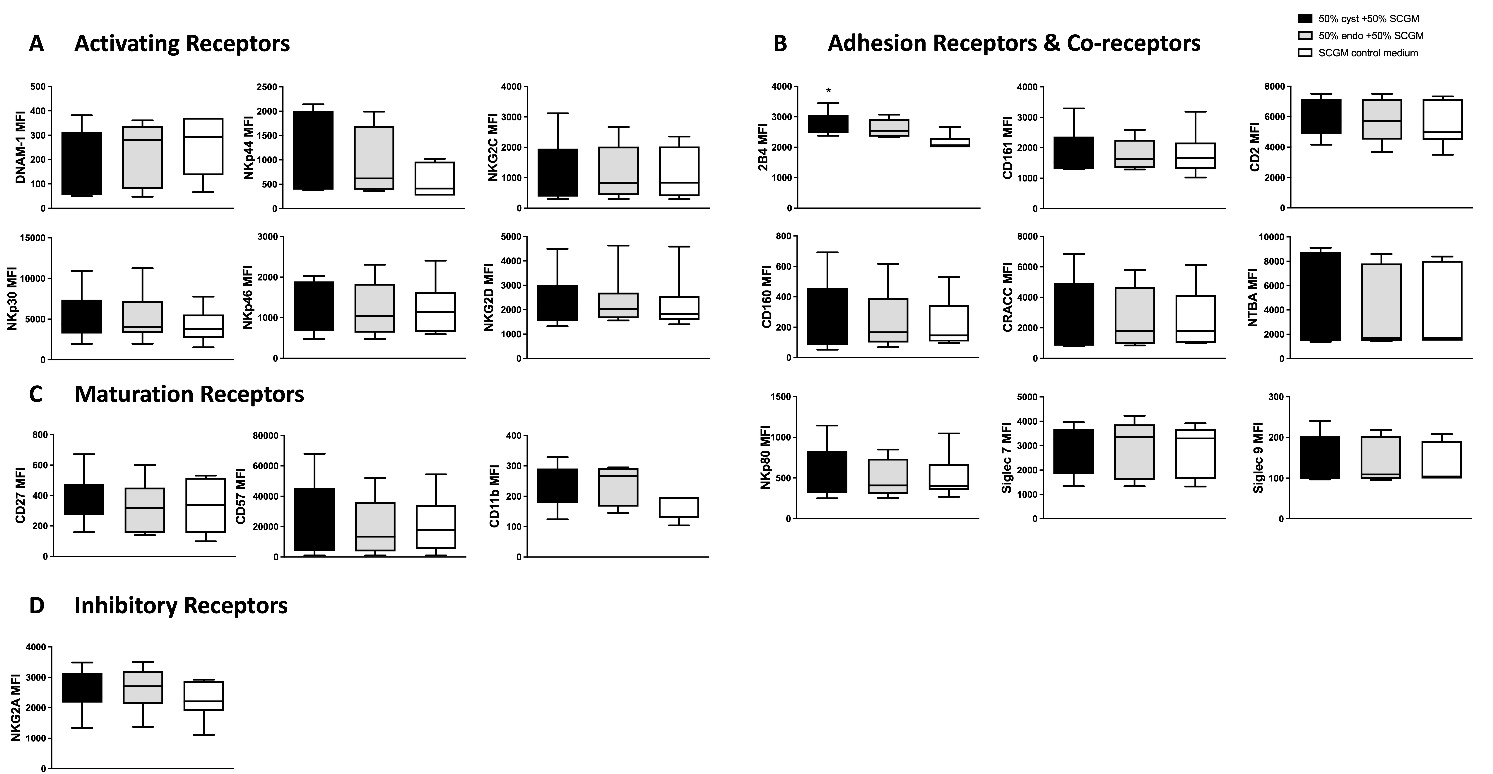
**Supplementary Figure 1 |** The phenotype of NK cells following culture in the conditioned medium of ESC_cyst_ or ESC_endo_, or in SCGM control medium for five days was analyzed using flow cytometry. There were no significant differences for the expression levels for all of the activating (A), adhesion receptors and co-receptors (B), and maturation (C) and inhibitory (D) receptors, between ESC_cyst_ and ESC_endo_-treated NK cells. The conditioned medium was 50% derived from ESC_cyst_ or ESC_endo_ and 50% complete SCGM growth medium, or 100% SCGM control medium. Eight independent experiments (n=3 biological replicates) were carried out. Mean +/- SD.


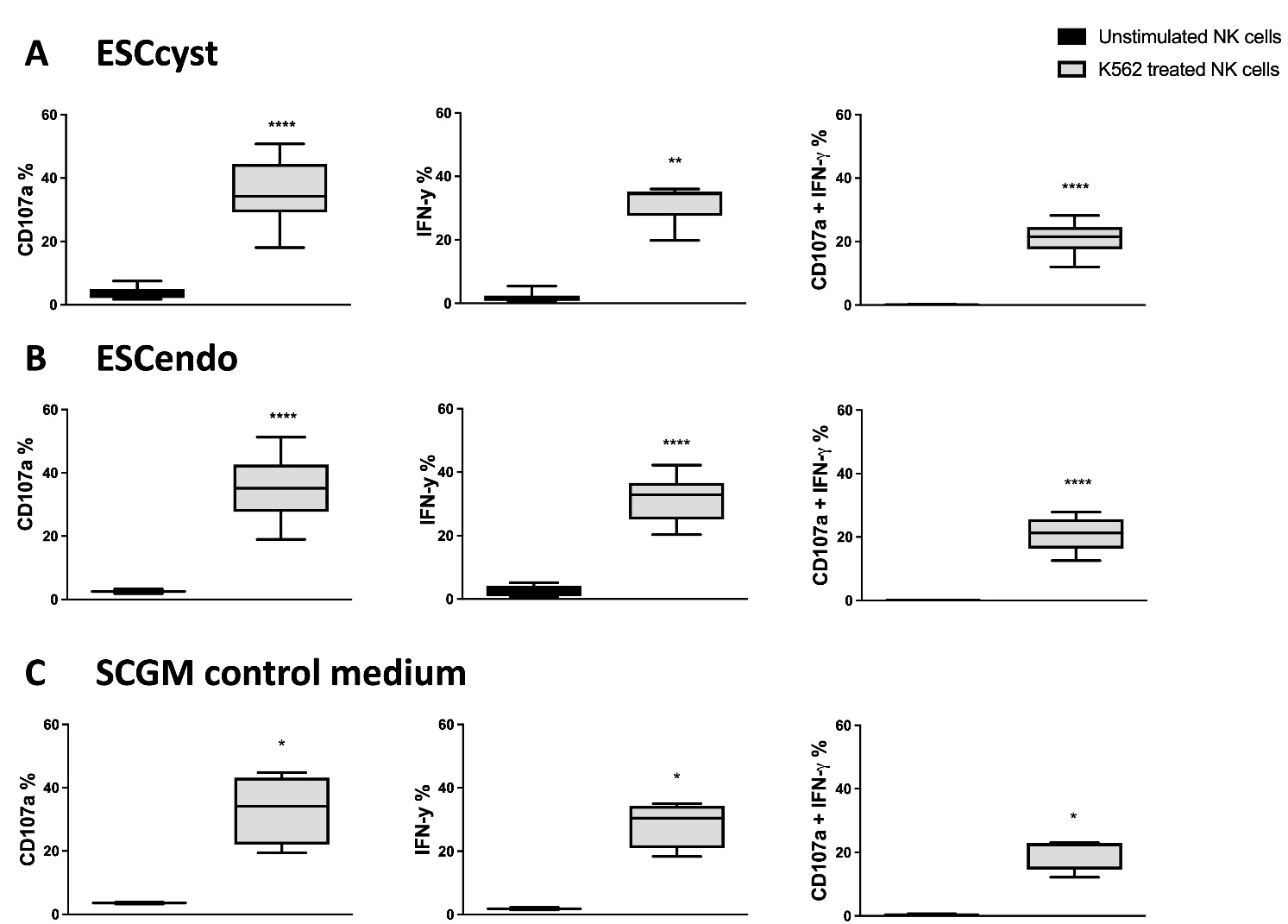


**Supplementary Figure 2 |** For the conditioned medium experiments, NK cells that were first cultured in the conditioned medium from ESC_cyst_, ESC_endo_ or in SCGM control medium then stimulated with the K562 cell line as a positive control showed that the percentage of NK cells that underwent degranulation (CD107a) or and had expression of IFN-γ was significantly (P < 0.05) greater compared to unstimulated NK cells (A, B and C). Six independent experiments were carried out. Mean +/- SD.


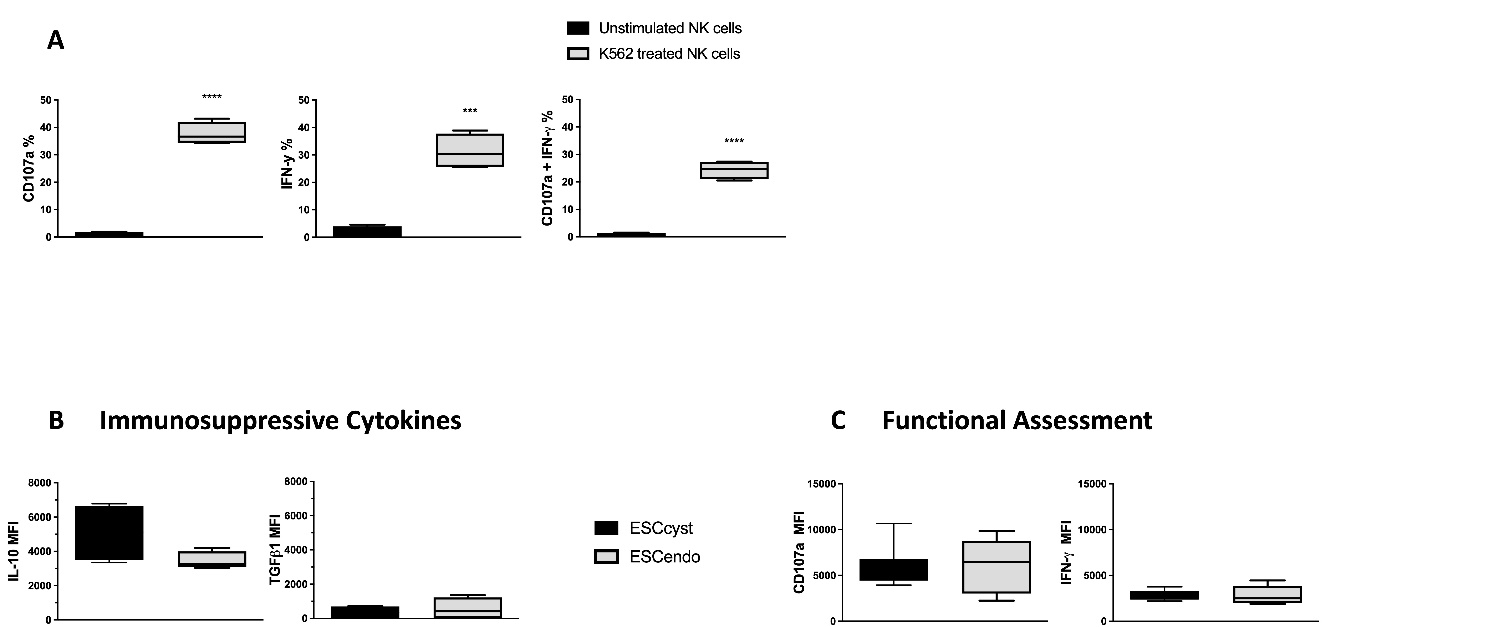
 **Supplementary Figure 3 |** Showing NK cells that were stimulated with K562 cells as a positive control for the ESC target cell killing experiments. Also, showing the phenotype and degranulation of NK cells stimulated with ESC_cyst_ or ESC_endo_ for four hours at a 3:1 cell ratio was analyzed using flow cytometry. The NK cells that were stimulated with the K562 cell line as a positive control showed that the percentage of NK cells that underwent degranulation (CD107a) or and had expression of IFN-γ was significantly (P < 0.05) greater compared to unstimulated NK cells (A). The expression levels of the immunosuppressive cytokines TGFβ1 and IL-10 in the NK cells that were stimulated with ESC_cyst_ were not different from the NK cells that were stimulated with ESC_endo_ (B). There were no significant differences between NK cells stimulated with ESC_cyst_ or ESC_endo_._._ Four independent experiments were carried out (A). Six-eight independent experiments (n=4 biological replicates) were carried out. Mean +/- SD.


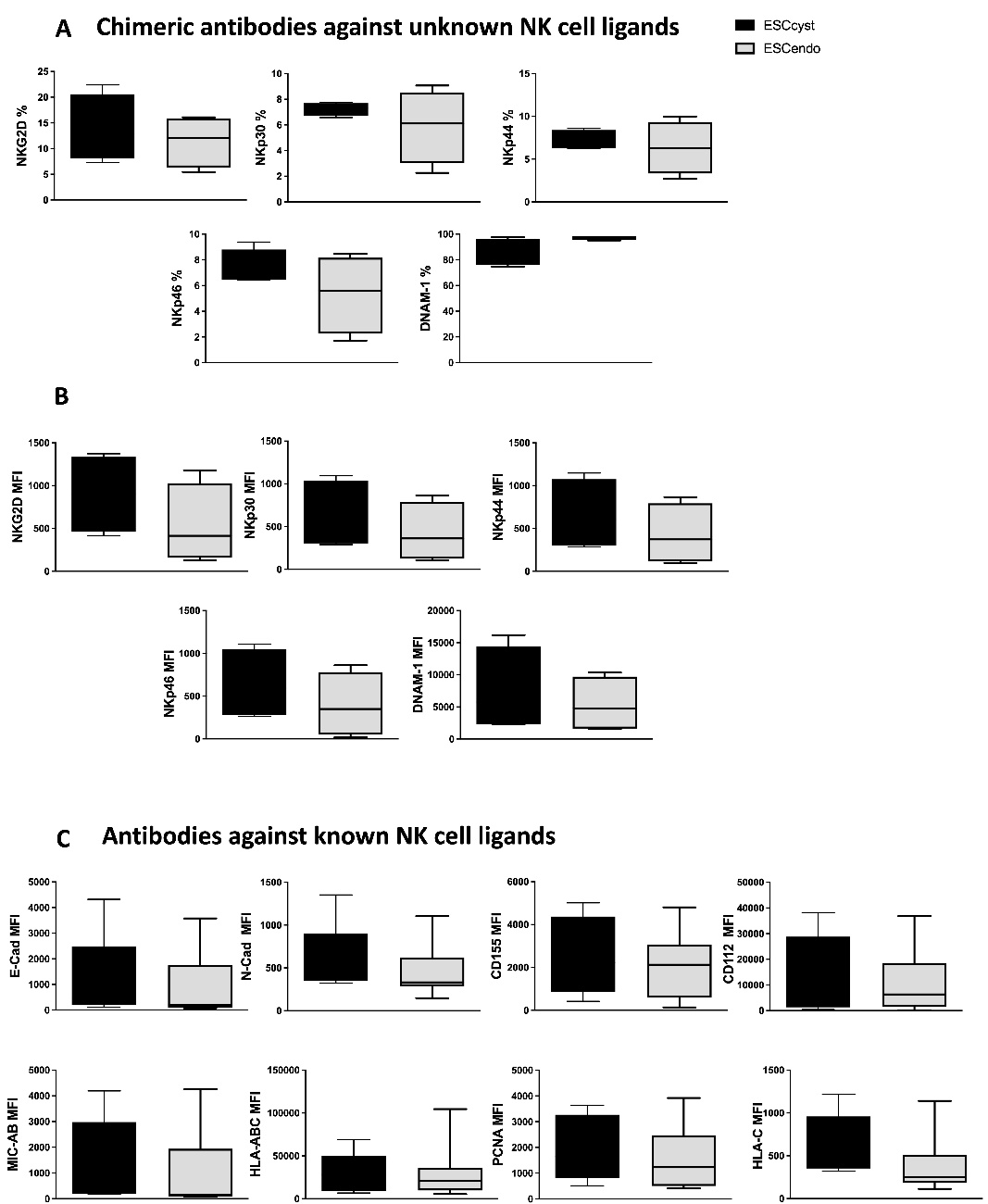


**Supplementary Figure 4 |** The expression of unknown and known NK cell ligands on ESC_cyst_ and ESC_endo_ was examined using chimeric and monoclonal antibodies. There were no differences between ESC_cyst_ and ESC_endo_ in the percentage expression and expression levels of any of the unknown NK cell ligands using the chimeric antibodies (A and B). There were no differences between ESC_cyst_ and ESC_endo_ in the expression levels of any of the known NK cell ligands using the monoclonal antibodies (C). Eight independent experiments (n=4 biological replicates) were carried out. Mean +/- SD.


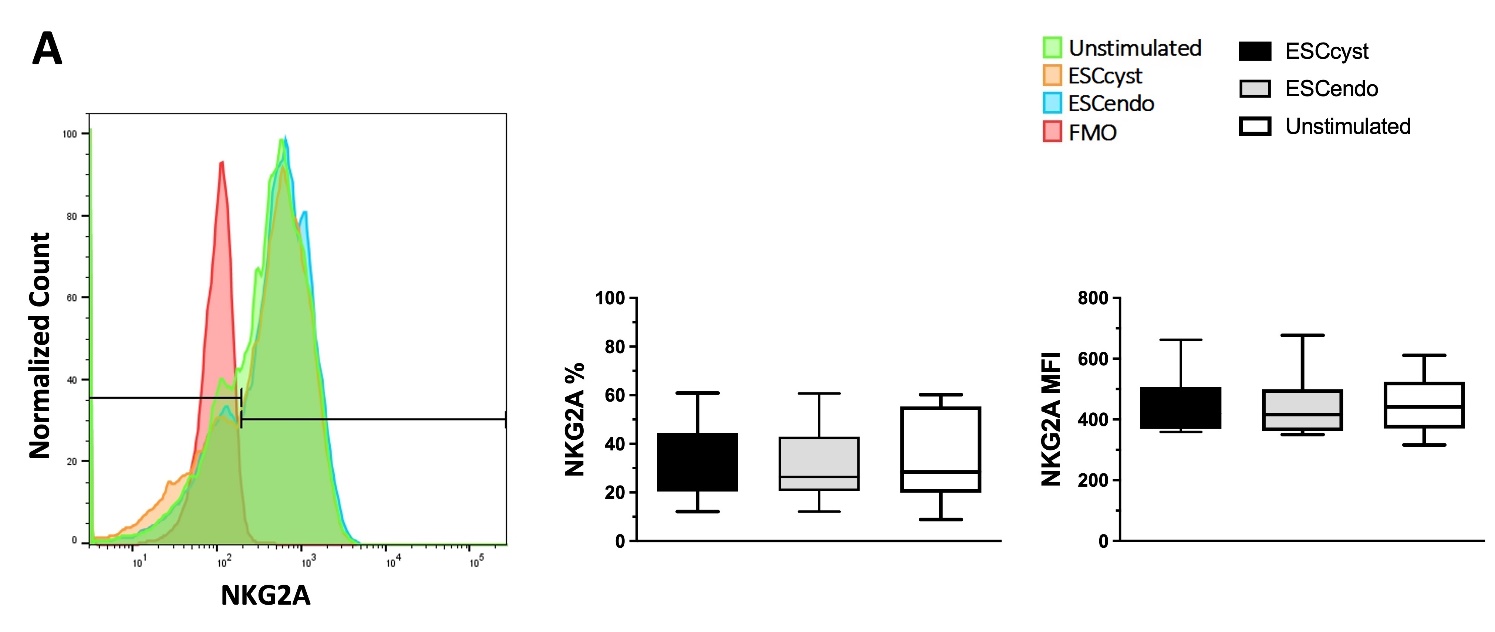
**Supplementary Figure 5 |** The expression of NKG2A in the NK cells stimulated with ESC_cyst_ or ESC_endo_ for four hours at a 3:1 cell ratio was analyzed using flow cytometry, showing a representative histogram. The percentage expression and expression levels of the surface inhibiting receptor, NKG2A, in NK cells that were stimulated with ESC_cyst_ was not significantly different compared to the NK cells stimulated with ESC_endo_. Unstimulated NK cells were incubated with medium only. Fluorescence minus one (FMO) control contained all of the NK cell markers examined, except NKG2A. Six-eight independent experiments (n=4 biological replicates) were carried out. Mean +/- SD.
